# Editorial: Unravelling the role of CB2r in neuropsychiatric diseases

**DOI:** 10.3389/fpsyt.2023.1171959

**Published:** 2023-03-20

**Authors:** Emmanuel S. Onaivi

**Affiliations:** Department of Biology, College of Science and Health, William Paterson University, Wayne, NJ, United States

**Keywords:** endocannabinoidome, CB2r, biomarker, neurodegenerative disorders, neuropsychiatric disorders

The explosion of technological advances and transformation of cannabinoid research drives the increasing global use and deregulation of cannabis and cannabinoids for medicinal and recreational purposes into mainstream science (1). This has revealed an expanded endocannabinoid system (ECS) known as “Endocannabinoidome,” (eCBome), which includes current and numerous lipid mediators, endocannabinoids (eCBs), enzymes, and receptors (2). This emerging ECS provides a platform for targeting components of the eCBome in neuropsychiatric disorders. Cannabinoid type 1 receptor (CB1R), type 2-cannabinoid receptor (CB2R), and other putative cannabinoid receptors (CBRs) are activated by eCBs, natural or synthetic cannabinoids, and other ligands for transient receptor potential channels (TRPs) and peroxisome proliferator-activated receptors (PPARs). These advances have now unraveled and demonstrated the functional expression of CB2Rs in neuronal, endothelial cells, and glial cells beyond the previous technological limitations when CB2Rs were undetectable in neurons and were called peripheral CB2Rs (1, 3–6). The concept of CB2R as peripheral CBRs that was thought to be predominantly expressed in immune cells was challenged, as many studies now indicate neuronal, glial, and endothelial cell expression of CB2Rs beyond immuno-cannabinoid activity (1, 3–5). Thus, neuroinflammation, an indicator and modulator of neurodegeneration, is emerging as a key component underlying the effects of CB2Rs that are associated with immune responses. Therefore, CB2Rs could be considered targets for the reduction of neuroinflammatory processes, the reduction of M1 neurotoxic state, and the induction of M2 neuroprotective state (7).

While there exists some controversy, ambiguity, and lingering debate about the functional neuronal expression of CB2Rs, a number of studies demonstrate that CB2Rs are expressed at low basal levels in the CNS, but that they are dynamic, inducible, and can be upregulated in neuropsychiatric and neurological disorders (2, 3, 8). This provides a potential role of CB2Rs in neuropsychiatric and neurodegenerative disorders, as CB1R ligands are associated with CNS side effects. Moreover, progress in the treatment of neuropsychiatric disorders has been limited, but novel approaches in genetic and epigenetic techniques with imaging of signaling in brain circuits are contributing to unraveling new biomarkers and therapeutic targets for neuropsychiatric disorders. The concerns and probable limitations for the use of CB2R ligands in neuropsychiatric disorders could be associated with peripheral side effects where they are expressed abundantly. However, it is noteworthy that the determination of CB1R and CB2R crystal structures, along with the quantum and chemometric methods to construct a partial least squares model may be used to design and generate compounds binding to both CB1R and CB2R in a therapeutic way without psychoactivity (9).

This is a special Research Topic and contribution of studies, “*Unravelling the role of CB2r in neuropsychiatric diseases*.” Advances on the role of CB2Rs in preclinical and clinical research studies are presented, including two review articles and three clinical research studies, focusing not only on CB1Rs and CB2Rs but also on the components of the eCBome studies in neurodegenerative and neuropsychiatric diseases. The shifting landscape and increasing new knowledge on cannabis/cannabinoid medicalization and recreational use provided an opportunity for Ishiguro et al. to review and summarize the potential role of CB2Rs in neuropsychiatric and neurodegenerative disorders. The review focused on CB2Rs that present therapeutic advantages over the CB1Rs that are associated with a number of physiological effects, including psycho-activity that limits their use. The initial excitement of targeting CB1R antagonist as an anti-obesity drug was withdrawn due to severe side effects, including suicides. In their review, Morcuende et al. provided a deeper overview of the “*Immunomodulatory role of CB2Rs in emotional and cognitive disorders*” with an insight into the emerging eCBome and microbiome crosstalk revealing gut-brain communication. This is because a number of studies show the involvement of CB2Rs in the immune system and the regulation of inflammation. Interestingly, the detection of CB2Rs in different brain areas including different brain cells, particularly in glia cells in the microglia, astrocytes, and neurons, supports the immunomodulatory role of CB2Rs in the immune system and brain circuitry associated with the pathophysiology of neurodegenerative and neuropsychiatric diseases. There are, however, limitations and concerns regarding the use of CB2R medication development for neurological and neuropsychiatric diseases as they are abundantly expressed in the periphery and may have peripheral side effects, while they may be useful in neurological and neuropsychiatric diseases associated with neuroinflammation. With the increasing evidence that CB2Rs are involved in the brain, the functional role of CB2R-eCBome neuro-immune axis in pathophysiological signaling in the development of neuropsychiatric diseases, therefore, warrants further investigation.

Of the three original research articles, data was presented on “*Cell-type specific deletion of CB2Rs in dopamine neurons induced hyperactivity phenotype: Possible relevance to attention-deficit hyperactivity disorder (ADHD)*” by Canseco-Alba et al.. This preclinical study using cell-type specific deletion of CB2Rs from dopamine neurons in the DAT-*Cnr2* and from microglia cells in the *CX3Cr1-Cnr2* conditional knockout (cKO) mice provided an unprecedented discovery and model on the role of CB2Rs in the CNS. First, the data showed that CB2Rs are found in dopamine neurons, and when deleted, the DAT-*Cnr2* animals exhibit continuous spontaneous hyperactivity compared to the wild type (WT) and the CX3Cr1-*Cnr2* cKO mice. When treated with amphetamine, this locomotor hyperactivity was reduced in the DAT-*Cnr2* cKO hyperactive mice; whereas, there was an increase in the WT control mice treated with the same low dose of amphetamine. Paradoxically, the first-line treatment for individuals with ADHD is with amphetamine medications that increase activity in individuals without ADHD but decrease activity in ADHD individuals. While mice are not humans, this paradoxical discovery in mice with DAT-*Cnr2* cKO mice was proposed as a possible model for studying medications that may be useful in ADHD. One of the clinical original research papers by Kang et al. reported the study on components of the eCBome relating to “*Loneliness, circulating endocannabinoid concentrations, and grief trajectories in bereaved older adults: a longitudinal study*.” Using a baseline clinical assessments loneliness scale, which was collected over 6 months, they reported that both eCBs, anandamide (AEA), and 2-arachidonly glycerol (2-AG) were higher in lonely grieving elders than in healthy controls. It was concluded that increase-circulating eCBs are associated with better adaptation to bereavement and that “circulating eCBs as potential moderators and mediators of the loneliness-grief trajectory should be investigated.” Finally, an exploratory clinical study by Soundararajan et al. investigated “*FAAH and CNR1 polymorphisms in the endocannabinoid system and alcohol-related sleep quality*” in patients with alcohol use disorder (AUD) and controls without AUD. In their study, *CNR1* and *FAAH* genetic variants were investigated in individuals undergoing inpatient treatment for AUD and non-treatment-seeking individuals along with control participants without AUD. They examined a gene set associated with ECS associated with sleep phenotypes in AUD, and they reported associations between *CNR1*/*FAAH* polymorphism and sleep quality in alcohol-associated sleep disturbances.

The significant progress and advances in the expanded ECS, the eCBome, is unraveling the molecular basis not only of the *CNR2* genomic structure, its polymorphic nature, sub-type specificity, and variations (SNPs and CNVs) but also of the components of eCBome. This offers opportunities for continued studies and clinical trials targeting the microbiome and elements of eCBome crosstalk that is revealing a bidirectional gut-brain communication in neuropsychiatric disease.

## Author contributions

EO: wrote, authenticated, and critically endorsed the editorial for publication.
